# Landscape-induced spatial oscillations in population dynamics

**DOI:** 10.1038/s41598-021-82344-8

**Published:** 2021-02-10

**Authors:** Vivian Dornelas, Eduardo H. Colombo, Cristóbal López, Emilio Hernández-García, Celia Anteneodo

**Affiliations:** 1 Department of Physics, PUC-Rio, Rua Marquês de São Vicente, 225, Rio de Janeiro, 22451-900 Brazil; 2 IFISC (CSIC-UIB), Campus Universitat Illes Balears, 07122 Palma de Mallorca, Spain; 3 Department of Ecology and Evolutionary Biology, Princeton University, Princeton, NJ 08544 USA; 4 Department of Ecology, Evolution, and Natural Resources, Rutgers University, New Brunswick, NJ 08901 USA; 5 Institute of Science and Technology for Complex Systems, Rio de Janeiro, Brazil

**Keywords:** Nonlinear phenomena, Biological physics, Population dynamics

## Abstract

We study the effect that disturbances in the ecological landscape exert on the spatial distribution of a population that evolves according to the nonlocal FKPP equation. Using both numerical and analytical techniques, we characterize, as a function of the interaction kernel, the three types of stationary profiles that can develop near abrupt spatial variations in the environmental conditions vital for population growth: sustained oscillations, decaying oscillations and exponential relaxation towards a flat profile. Through the mapping between the features of the induced wrinkles and the shape of the interaction kernel, we discuss how heterogeneities can reveal information that would be hidden in a flat landscape.

## Introduction

The Fisher–Kolmogorov–Petrovskii–Piskunov (FKPP) equation^1–3^ is the standard model describing, at a continuum level, the spatio-temporal dynamics of a population of individuals that diffuse, grow and compete for resources. In one dimension, it is given by

1 $$\begin{aligned} \partial _t \rho (x,t) = D\partial _{xx} \rho (x,t) + a\rho (x,t)-b\rho ^2(x,t)\, , \end{aligned}$$

where $\rho (x,t)$ is the population density at position *x* and time *t*, *D* is the diffusion coefficient, *a* is the (clonal) reproduction rate, and *b* is the strength of (intraspecific) competition that bounds population growth.

In Eq. (1) competition is local, in the sense that it occurs at scales much smaller than those associated with the diffusion process. However, competition processes might also extend to larger scales. This can be promoted by the underlying dynamics of interaction mediators (e.g., shared resources), such that even if individuals’ actions are locally initiated, the effects of these actions propagate to the surroundings. Along the lines of the Turing mechanism^4^, the mediator dynamics can be explicitly modeled using an additional reaction–diffusion equation, an approach that has been widely applied to water-vegetation systems^5–9^. Also in the context of vegetation dynamics, competition among plants can be mediated by roots^10,11^, that extend beyond the surface vegetation. More generally, spatially-extended interactions between many types of organisms can be generated by various other mechanisms, such as acoustic communication^12^, exchange of physico-chemical signals^13–16^, and spatial exploitation, as present in the case of sensile^17^ and territorial^18^ organisms.

**Figure 1:**
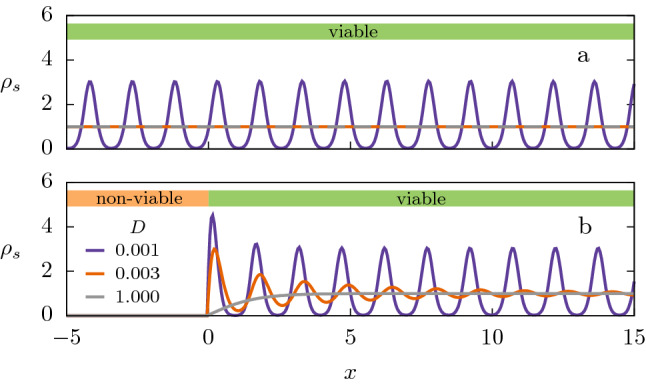
Population distribution in a medium which is (**a**) homogeneously viable, (**b**) heterogeneous, with viable and non-viable regions. Depending on the values of the parameters in Eq. (3), spatial patterns can develop around the uniform steady state in (**a**), and they are preserved in the viable region of the corresponding case in (**b**). But even when the steady state is uniform in (**a**), decaying oscillations can emerge in (**b**). Parameters are $a=b=1$, values of *D* are given in the legend, and for kernel $\gamma _q$ defined in Eq. (4), we fix $q=-0.5$ and $\ell =2$. For panel (**b**), *A* in Eq. (9) is $A \rightarrow \infty$.

Although the intraspecific competitive interaction is established by other species or substances that act as mediators, when their timescales are much shorter than the population ones, a single equation for the distribution of individuals can be derived^19^. This effective equation contains a nonlocal term describing the influence of individuals at a distance. Due to the often complex web of processes regulating the mediator dynamics, a useful phenomenological approach is to incorporate their effects with an interaction kernel (also called influence function) $\gamma (x)$, describing how the effective interaction between individuals decreases with their distance *x*. Furthermore it allows to address rather generally the impact of distance-dependent competition regardless of the mechanisms behind it. Then, Eq. (1) is extended as^20–22^

2 $$\begin{aligned} \partial _t \rho (x,t) = D\partial _{xx}\rho (x,t) + a\rho (x,t) - b \rho (x,t)[\gamma \star \rho ](x,t) \,, \end{aligned}$$

where $[\gamma \star \rho ](x,t) \equiv \int _{-\infty }^{\infty }\gamma (x-x')\rho (x',dt) dx'$, and $\int _{-\infty }^{\infty }\gamma (x)dx=1$. The particular shape and characteristic scales of $\gamma$ effectively embody the details of the interaction mechanisms. At a difference from the original FKPP, the nonlocal FKPP equation, given by Eq. (2), can exhibit self-organized structures (as depicted in Fig. 1a) depending primarily on the particular properties of the kernel and, secondarily, on the values of the diffusion and reproduction rates^21–24^. This is a minimal continuous-field description of population of individuals that compete nonlocally, containing the essential ingredients used to model diverse species dynamics^22^.

Moreover, Eq. (2) assumes a homogeneous environment, which is implicit in the constant coefficients. But, actually, in biological systems, environmental factors suffer spatial variations^25–27^. In this paper, we exploit that they can stress the system and resonate with the internal scales^20,28–31^, generating spatial oscillations in the distribution of the population that can serve to unveil hidden information. In order to do that, we consider the following extension of Eq. (2),

3 $$\begin{aligned} \partial _t \rho (x,t) = D\partial _{xx}\rho (x,t) + \Psi (x)\rho (x,t) - b \rho (x,t)[\gamma \star \rho ](x,t)\,, \end{aligned}$$

where the spatially-dependent reproduction rate, $\Psi (x)$, reflects the overall habitat quality at a given location *x*^27^.

Particular forms of $\Psi$, accounting for diverse complex spatiotemporal features of natural environments, have been considered in previous studies^20,32–34^. They have shown how this spatial dependence can modify the stability domains or even generate new states that were absent otherwise. For the particular case in which environmental disturbances are random, these results can be framed in the context of *noise-induced transitions*^31,35,36^.

In this work, we focus on sharp changes in the spatial environmental conditions relevant for the population under consideration^26,27,37^. This kind of change is found in diverse situations in nature, e.g., at the interface between forest and grassland^27^, at the bounds of oases^25^ or harmful regions^38^, or in artificial lab experiments^26^, where there is a neat contrast of spatial domains with different growth rates. Contemplating these cases justifies attributing the Heaviside step or rectangular functions to $\Psi (x)$. For Turing-like models, it has been shown that the existence of a step-function hetereogenity can promote the formation of decaying oscillations even when the system is stable under homogeneous conditions^34^. We extend this discussion by noting that there is a close relation between the landscape-induced states and the underlying dynamics of the interaction mediators which, in our case, is captured by the influence function.

We perform a systematic exploration of the model parameter space and investigate the emergence of the three kinds of stationary (long time) population profiles that can develop from the interface between regions of contrasting characteristics: *sustained oscillations* (or spatial patterns, without amplitude decay), *decaying oscillations* (with decreasing amplitude from the interface) or *exponential decay* towards a flat profile. These behaviors are schematically depicted in Fig. 1b. Ultimately, we report the existence of a one-to-one mapping between the influence function parameters and the oscillations features, allowing us to extract details of the interaction from the pattern images^39^.

The paper is organized as follows. In Preliminaries section, we provide introductory information with general considerations about the homogeneous environment as a frame of reference. In Heterogeneous landscapes section, we present the main results for 1D landscapes with sharp changes. Additionally, outcomes for 2D landscapes are displayed. In Inferring information about the interaction section, we discuss how information about the interaction kernel can be extracted from observable oscillations. A summary of the main findings and discussion are presented in Final remarks section.

## Preliminaries

In this section, we first define the main class of influence functions that will be used in numerical examples throughout the paper. We also revisit the linear response analysis for the homogeneous environment, which serves as a reference frame for the more complex heterogeneous case.

### Interaction kernel

**Figure 2:**
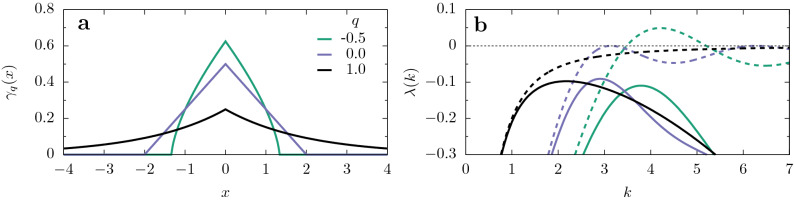
Interaction kernel and mode stability in a homogeneous medium. (**a**) $\gamma _q(x)$, defined in Eq. (4), for the values of *q* indicated on the figure, and $\ell =2$. (**b**) Mode growth rate $\lambda (k)$ given by Eq. (5), for $a=b=1$, with $D=0$ (dashed lines) and $D=0.01$ (solid lines), corresponding to the values of *q* plotted in (**a**). The case $q=0$ (triangular kernel) is the critical one, for which the maximal value of $\lambda (k)$ at finite *k* is zero when $D=0$. Notice that, when diffusion is absent, the mode growth rate is proportional to the kernel Fourier transform (see Eq. (5)).

We have chosen a family of influence functions that allows us to continuously vary its compactness:

4 $$\begin{aligned} \gamma _q(x) = N_q [1 - (1-q)|x|/\ell ]_+^{1/(1-q)} \,\equiv \, N_q \exp _q(|x|/\ell ) \, , \end{aligned}$$

where *q* and $\ell$ control the shape and scale of the kernel, respectively, and $N_q$ is a normalization constant. The subindex + means $[z]_+=z$, if $z > 0$, and $[z]_+=0$ otherwise. This kernel is based on a generalization of the exponential function, known as *q*-exponential^40^. In the limit $q\rightarrow 1$, the standard exponential is approached yielding $\gamma _1(x) \propto e^{-|x|/\ell }$. The kernel shapes, for different values of *q* are illustrated in Fig. 2a. As we will see, it is specially relevant the fact that, only for $q<0$, the Fourier transform of $\gamma _q(x)$ can take negative values. Then, we focus on the range $-1 \le q <1$, around this critical value. Moreover, in this range, the interaction is restricted to a finite region and the kernel moments are well-defined, a fact that will facilitate both the numerical and theoretical approaches. The family of stretched exponential kernels was also considered for comparison (see Supplementary Information).

### Homogeneous landscapes

For a homogeneous landscape, with $\Psi (x)=a$, the linearization of Eq. (3) around its uniform solution $\rho _0=a/b$, done by setting $\rho (x,t) = \rho _0 + \varepsilon (x,t)$ (with $\varepsilon /\rho _0 \ll 1$), gives $\partial _t\tilde{\varepsilon }(k,t)= [-Dk^2 - a\tilde{\gamma }(k)]\tilde{\varepsilon }(k,t)$ in Fourier space, where $\tilde{\gamma }(k) = \int _{-\infty }^{\infty } \gamma (x) e^{- i k x} dx$ is the Fourier transform of the interaction kernel $\gamma$. The factor between square brackets represents the growth rate of mode *k*,

5 $$\begin{aligned} \lambda (k) = -Dk^2 - a\tilde{\gamma }(k)\, , \end{aligned}$$

which, for the considered kernels, is a real function, and whose shape is plotted in Fig. 2b, for each kernel $\gamma _q$ shown in Fig. 2a. It is the important quantity that will appear all throughout the paper, since solutions of the transformed linearized equation satisfy $\tilde{\varepsilon }(k,t)= \tilde{\varepsilon }(k,0) \mathrm{e}^{\lambda (k)t}$. Thus, if $\lambda (k)<0$ for all *k*, any initial perturbation will fade out, such that in the long-time limit the population distribution, $\rho (x)$, will be flat. On the contrary, if there are unstable modes, with $\lambda (k)>0$, stationary spatial oscillations will be produced with a characteristic mode $k^\star$ (the maximum of $\lambda$), which is the initially fastest growing one^41^.

From Eq. (5), $\lambda (k)>0$ occurs for sufficiently small *D* if the Fourier transform of the kernel takes some negative values. Then, by substitution of $\tilde{\gamma }_q$ into Eq. (5), we conclude that sustained oscillations can only appear if $\gamma _q(x)$ is sub-triangular, i.e., $q<0$ (in the critical case $q=0$, $\gamma _q(x)$ produces the triangular kernel, whose Fourier transform is $\tilde{\gamma }_0(k)= \sin ^2(k \ell )/( k \ell )^2$). This is a necessary but not sufficient condition that arises by imposing $\lambda (k^\star )>0$ in the most favorable case $D=0$ (hence $\tilde{\gamma }(k^\star )<0$), to induce the growth of certain modes. In contrast, for $q \ge 0$, the uniform state is intrinsically stable (that is, independently of the remaining parameters). In Fig. 2b, we plot the mode growth rate for $D=0$ and $D>0$, which shows how diffusion affects mode stability, damping inhomogeneities in the population distribution.

Concerning the interaction length $\ell >0$, when $D=0$, it simply scales the wavenumber as $k\ell$. Therefore, when $\ell$ goes to zero (implying local dynamics), $\lambda (k) \rightarrow \lambda (0)<0$, meaning that patterns go continuously to a flat profile in that limit. In contrast, for $D>0$, the first term in Eq. (5) has a more homogenizing effect the larger is $k^\star$, hence the smaller is $\ell$. As a consequence, despite interactions are nonlocal, patterns emerge only for $\ell$ above a critical value^41^. For $D>0$, there is also a critical reproduction rate, $a_c$, such that sustained oscillations emerge only for $a>a_c$.

In summary, in the cases where $\lambda (k^\star ) \le 0$, i.e., either $q \ge 0$, or $q < 0$ with sufficiently large *D* (or, alternatively, small enough $\ell$ or *a*), information regarding the interaction scale $\ell$ or other details of the kernel profile are not stamped in the spatial distribution $\rho (x,t)$, which becomes uniform at long times.

## Heterogeneous landscapes

In this section, the heterogeneity of the landscape is introduced by assuming that its profile can be written as $\Psi (x) = a + \psi (x)$, where $\psi (x)$ represents the spatial variations of the environment around a reference level *a*.

The results that we will present were obtained through theoretical and numerical techniques. The theoretical approach is based on the mode linear stability analysis discussed in the previous section. Numerical integration of Eq. (3), starting from a homogeneous state plus a random perturbation, was performed following an explicit forward-time-centered-space scheme, with boundary conditions suitably chosen for each case (see Supplementary Information for details).

### Refuge

As a paradigm of a heterogeneous environment with sharp borders, we first consider that the spatial variations around the reference level *a* are given by

6 $$\begin{aligned} \psi (x) = - A[1- \Theta (L/2 -|x|)] \,, \end{aligned}$$

where $\Theta$ is the Heaviside step function. With $A>0$, it represents a refuge of size *L* with growth rate *a* immersed in a less viable environment with growth rate $a-A$. In a laboratory situation, this can be constructed by means of a mask delimiting a region that protects organisms from some harmful agent, for instance, shielding bacteria from UV radiation^26^. In natural environments, this type of localized disturbance appears due to changes in the geographical and local climate conditions^27^, or even engineered by other species^38^.

**Figure 3:**
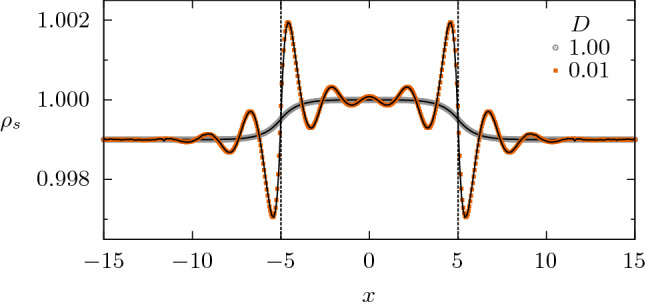
Stationary population density $\rho _s$ vs. *x* in a refuge. This heterogeneous environment is defined by Eq. (6), with $a=b=1$, $A=10^{-3}$ and $L=10$. The vertical lines indicate the refuge boundaries. We used the kernel $\gamma _q(x)$, with $q=0.1$ and $\ell =2$, and two different values of *D*. Symbols are results from numerical integration of Eq. (3) under periodic boundary conditions, and solid lines from the small-*A* approximation given by Eq. (8), in excellent agreement with the exact numerical solution. Recall that, in a homogeneous environment, no oscillations appear for $q \ge 0$.

In Homogeneous landscapes section, we have seen that the uniform distribution is intrinsically stable when $q \ge 0$. In contrast, when there are heterogeneities in $\Psi (x)$, spatial structures can emerge even if $q\ge 0$, as illustrated in Fig. 3 for the case $D=0.01$.

In the limit of weak heterogeneity, i.e., under the condition $|\psi (x)|/a \ll 1$, we obtain an approximate analytical solution assuming that the steady solution of Eq. (3) can be expressed in terms of a small deviation $\varepsilon _s(x)$ around the homogeneous state $\rho _0=a/b$. Then, we substitute $\rho _s(x)=\rho _0+\varepsilon _s(x)$ into the stationary form of Eq. (3), discard terms of order equal or higher than $\mathcal{O}(\varepsilon ^2, A\varepsilon ,A^2)$, and Fourier transform, obtaining

7 $$\begin{aligned} \tilde{\varepsilon }_s(k) = \dfrac{ \rho _0 \tilde{\psi }(k)}{-\lambda (k)}\,, \end{aligned}$$

where $\lambda (k)$ was already defined in Eq. (5) and $\tilde{\psi }(k)$ is the Fourier transform of the small fluctuations in the landscape quality, which for the case of Eq. (6) is $\tilde{\psi }(k)= A[2\sin (Lk/2)/k -2\pi \delta (k)]$.

Finally, assuming that $\lambda (k^\star )<0$, the steady density distribution is given by

8 $$\begin{aligned} \rho _s(x) \,=\, \rho _0 +\varepsilon _s(x) \,=\, \rho _0 + \mathcal{F}^{-1}\Bigl (\dfrac{ \rho _0 \tilde{\psi }(k)}{-\lambda (k)}\Bigr ) \,, \end{aligned}$$

where the inverse Fourier transform $\mathcal{F}^{-1}$ must be numerically computed in general. For small heterogeneity, Eq. (8) is in very good agreement with the exact numerical solution obtained by integration of the dynamics Eq. (3), as can be seen in Fig. 3. Notice the two different profiles, depending on the diffusion coefficient *D*: one gently following the landscape heterogeneity and the other strongly oscillatory.

For small *D*, the induced oscillations display two evident characteristics, which depend on $\tilde{\gamma }_q$: a well-defined wavenumber and an amplitude that decays with the distance from the interface at $x=\pm L/2$ (highlighted by dashed vertical lines in Fig. 3). We will see in the next section how the characteristics of the oscillations reflect the details of the kernel $\gamma _q$.

### Semi-infinite habitat

Since oscillations are induced by changes in the landscape, it is worth focusing, from now on, on one of the interfaces between a more viable region and a less viable one. Moreover, we assume a refuge much larger than the oscillations wavelength, sufficient to follow over several cycles the structure originated at the interface. To do that, we consider a semi-infinite habitat defined by

9 $$\begin{aligned} \psi (x) = -A\Theta (-x) \,, \end{aligned}$$

where for convenience the interface was shifted to $x=0$, such that the low-quality region is at $x<0$. As an additional feature, we consider that the harmful conditions are very strong, that is, $A \rightarrow \infty$. The purpose is twofold, on the one hand, it allows to test the robustness of the results beyond the small-*A* approximation, on the other, it allows a simplification as follows. When $A \gg a$, $\rho$ is very small in the unfavorable region, then the nonlinear competition term in Eq. (3) can be neglected, leading to a steady distribution that decays exponentially from the interface as $\rho (x<0) \sim \exp [\sqrt{(A-a)/D}\,x]$. Thus, in the limit $A\rightarrow \infty$, we have $\rho (x <0,t)= 0$. In addition, the semi-infinite habitat is simulated by the interval [0, *L*], where *L* ($=100$ in our simulations) is large enough in comparison to oscillation length-scales. Then, far away from the interface, we set $\rho (x \ge L,t)=\rho _0$. This is the setting used to produce Fig. 1b, by numerical integration of Eq. (3).

**Figure 4:**
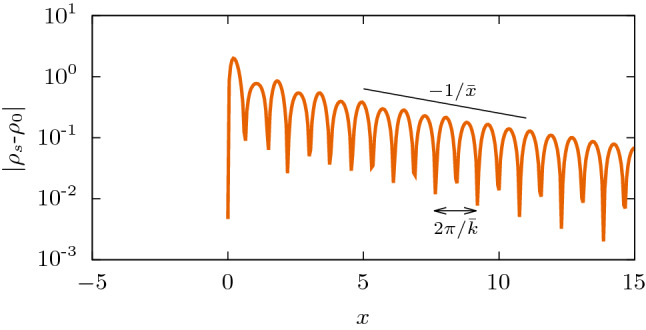
Characterization of stationary profiles. Long-time solutions approach a stationary state characterized by the wavelength $2\pi /\bar{k}$ and decay length $\bar{x}$. The slope of the straight black line is indicated in the figure. This example was obtained from numerical integration of Eq. (3), assuming a semi-infinite habitat, with parameters $D= 0.003$, $\gamma _q(x)$ with $\ell =2$ and $q=-0.5$.

As sketched in Fig. 4, for each steady distribution attained at long times, we measure the wavelength, from which we obtain the wavenumber $\bar{k}$, and the decay length $\bar{x}$, by observing that the envelope of the oscillations decays as $\exp (-x/\bar{x})$.

**Figure 5:**
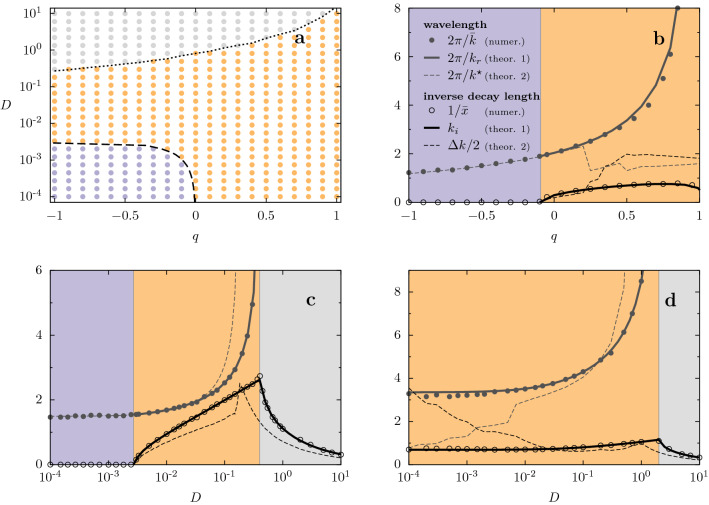
Phase diagram and characteristics of the stationary profiles as a function of diffusion coefficient *D* and *q*, in the semi-infinite habitat. We used the kernel $\gamma _q(x)$, with $\ell =2$. (**a**) Phase diagram in the $q-D$ plane, and cuts at (**b**) $D=10^{-3}$, (**c**) $q=-0.5$ (**d**) $q=0.5$. The remaining parameters are $a=b=1$. In diagram (**a**), for each point in the grid, the type of regime was determined based on the values of $2\pi /\bar{k}$ and $\bar{x}$ that characterize the solutions of Eq. (3): *sustained oscillations* ($\bar{k}>0$ and $\bar{x}\rightarrow \infty$, lilac), *decaying oscillations* ($\bar{k}>0$ and finite $\bar{x}$, orange), and *pure exponential decay* ($\bar{k}=0$ and finite $\bar{x}$, gray). In (**a**), the dashed and dotted lines correspond to ${k_i}=0$ and ${k_r}=0$, respectively, where ${k_r}$ and ${k_i}$ are the real and imaginary parts of the zeros of $\lambda(k)$, with the smallest positive imaginary part. In (**b**)-(**d**), symbols correspond to measurements of numerical profiles, according to Fig. 4, and solid lines correspond to the prediction in Eq. (10) (theoretical 1). Thin dashed lines correspond to the harmonic estimate (theoretical 2) given by Eq. (14).

The stationary spatial structures that emerge for $x>0$ can be classified into the three types depicted in Fig. 1: *sustained oscillations* (lilac line, with $\bar{k}>0$ and $\bar{x}\rightarrow \infty$); *decaying oscillations* (orange line, with $\bar{k}>0$ and finite $\bar{x}$); *exponential decay* (gray line $\bar{k}=0$ and finite $\bar{x}$). In the case of Fig. 1b, these three types appear when *D* changes. We also systematically varied the shape parameter *q* to construct the phase diagram in the plane $q-D$ presented in Fig. 5a.

To perform a theoretical prediction of $$\bar{k}$$ and $$\bar{x}$$, within the linear approximation, we consider that these oscillation parameters should be related to the poles of the integrand $$\mathrm{e}^{ikx} \tilde{\psi }(k) /[-\lambda (k)]$$ in the expression for the inverse Fourier transform that provides the solution, according to Eq. (8). As far as the external field $$\psi (x)$$ does not introduce non-trivial poles, like in the case of a Heaviside step function ($$\tilde{\psi }(k) \sim 1/k$$), only the zeros of the complex extension of $$\lambda (k)$$ matter. The dominant (more slowly decaying mode) is given by the complex poles $$k=\pm k_r + i k_i$$ $$ (k_r>0)$$ with minimal positive imaginary part that, except for amplitude and phase constants, will approximately provide patterns of the form $$\mathrm{e}^{-k_i x} \cos (k_r x)$$, allowing the identifications

10 $$\begin{aligned} \bar{k} = k_r\quad \text { and }\quad 1/\bar{x}= k_i. \end{aligned}$$

This theoretical prediction^42^ is in very good agreement with the results of numerical simulations, as shown in Fig. 5, explaining the observed regimes.

Moreover, the modes that persist beyond the interface have relatively small amplitudes, so that the system response is approximately linear in this region.

Lastly, recall that this analysis assumes mode stability ($\lambda (k)<0$). When $\lambda (k^\star )>0$, the system is intrinsically unstable, with the poles having null imaginary part (lying on the real axis). Nevertheless, the initially fastest growing mode, given by the maximum of $\lambda (k)$, tends to remain the dominant one in the long term^41^, yielding $\bar{k} \simeq k^\star$ for the sustained oscillations ($\bar{x} \rightarrow \infty$).

In order to obtain further insights, it is useful to consider the response function $\tilde{R}(k)$ that, from Eq. (7), is

11 $$\begin{aligned} \tilde{R}(k) \equiv \frac{|\tilde{\varepsilon }_s(k)|^2}{|\tilde{\psi }(k)|^2} = \frac{\rho _0^2}{\lambda ^{2}(k)} \,. \end{aligned}$$

Despite missing some of the dynamical information contained in the phase of $\lambda (k)$, it can provide a more direct estimation of the observed parameters than through calculation of the poles. In order to perform this estimation, we resort to the response function of a driven damped linear oscillator^43^ described by the equation $\varepsilon _H''(x)+2\zeta k_0\varepsilon _H'(x)+k_0^2\varepsilon _H(x)= f(x)$. We have

12 $$\begin{aligned} \tilde{R}_H(k) \equiv \frac{|\tilde{\varepsilon }_H(k)|^2}{|\tilde{f}(k)|^2}= \frac{1}{|\lambda _H(k)|^{2}} = \frac{1}{(k^2-k_0^2)^2+4\zeta ^2k_0^2k^2}\, , \end{aligned}$$

with $-\lambda _H(k) = -k^2 + i2\zeta k_0 k + k_0^2$, whose zeros (poles of $1/\lambda _H(k)$) are $k= \pm k_r + i k_i = k_0 (\pm \sqrt{1-\zeta ^2}+i\zeta )$, where $k_0$ is the natural mode and $\zeta$ is the damping coefficient. Note that, under a step forcing $f(x)=k_0^2 \Theta (x)$, which simulates our present setting, those poles carry the essential information of the damped-oscillation solution, given by $\tilde{\varepsilon }_H(k) = \tilde{f}(k)/[-\lambda _H(k)]$, where $\tilde{f}(k)=k_0^2(\pi \delta (k) -i/k)$. In the underdamped case ($\zeta <1$), this solution is explicitly given by

13 $$\begin{aligned} \varepsilon _H(x) = \left[ 1 - \frac{k_0}{\kappa } e^{-x/\xi } \sin (\kappa x +\phi )\right] \Theta (x) \, , \end{aligned}$$

where $\kappa = k_0\sqrt{1 -\zeta ^2}$ ($=k_r$), $\xi = 1/(\zeta k_0)$ ($=1/k_i$), and the phase constant $\phi =\tan ^{-1}(\xi \kappa )$. The solution for the overdamped case emerges for $\zeta >1$, when the zeros of $\lambda (k)$ are pure imaginary with $k_i=k_0(\zeta \pm \sqrt{\zeta ^2-1})$. The connection between the poles of $\tilde{R}_H(k)$ and the dynamic solution of the driven harmonic oscillator is possible because, as previously discussed, $\tilde{f}$ does not introduce relevant poles, and the forced solution has a form similar to the unforced one.

The harmonic model is, in fact, the minimal model sharing characteristics with our observed structures, and the correspondence between Eqs. (12) and  (13) will allow to estimate the oscillation features. In the limit of small $\zeta$, $\tilde{R}_H(k)$ has a sharp peak, characterized by a large quality factor $Q \equiv k^\star /\Delta k$, where $\Delta k$ is the bandwidth at half-height of $\tilde{R}(k)$ around $k^\star$^43^. First, we see that the position of the peak of $\tilde{R}_H$ approximately gives the oscillation mode $\kappa$, according to $k^\star = k_0\sqrt{1-2\zeta ^2} = \kappa + \mathcal{O}(\zeta ^2)$. Second, the bandwidth is related to the decay-length through $\Delta k = 2/\bar{x} + \mathcal{O}(\zeta ^2)$^44^.

Putting all together, as long as $\tilde{R}(k)$ resembles the bell-shaped form of $\tilde{R}_H(k)$, we can use the following estimates, which are correct for the harmonic case to first order in $\zeta$:

14 $$\begin{aligned} \bar{k} \simeq \underset{k}{\text {arg max}}\,(\tilde{R}) \equiv k^\star \quad \text { and }\quad \bar{x} \simeq \frac{2}{\Delta k} \,. \end{aligned}$$

The expression for $\bar{x}$ is also valid in the overdamped limit (large $\zeta$ in the harmonic model), in which case the maximum is located at $k^\star =0$.

**Figure 6:**
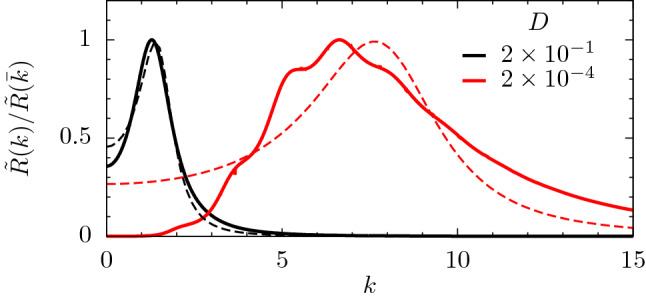
Comparison of $\tilde{R}(k)$ with the harmonic response $\tilde{R}_H(k)$, both normalized to their maximal values. $\tilde{R}(k)$ of our model, given by Eq. (11) (solid lines) and harmonic response $\tilde{R}_H(k)$, given by Eq. (12) (dashed lines), where the values of $k_0$ and $\zeta$ were obtained by fitting Eq. (12) to $\tilde{R}(k)$. In all cases, $q=0.5$, $\ell =2$ and two different values of *D* shown in the legend were considered. Notice that for $D=2\times 10^{-1}$, the response can be described by the harmonic approximation. For $D=2\times 10^{-4}$, the response is multipeaked, indicating that the harmonic approximation fails. In fact, the dominant mode observed in the simulations is not given by the absolute maximum, but by the small hump at $k\simeq 2.1$, as predicted by the analysis of complex poles.

The adequacy of the harmonic framework as an approximation to the response function of our model, $\tilde{R}(k)$, is illustrated in Fig. 6. In the case $D=2\times 10^{-1}$, the harmonic response is able to emulate $\tilde{R}(k)$. Then, if the harmonic approximation holds, one expects that the estimates given by Eq. (14) should work for the population dynamics case. In fact, they do work, as we will see below. Differently, when $D=2\times 10^{-4}$, $\tilde{R}(k)$ does not follow the harmonic shape, it is multipeaked and the dominant mode observed in the simulations is not given by the absolute maximum.

In Fig. 5, we compare the values of $\bar{k}$ and $\bar{x}$ extracted from the numerical solutions of Eq. (3) with those estimated by Eq. (14) (dashed lines) and, more accurately, with those predicted from the poles of $\tilde{R}(k)$ (solid lines), which perfectly follow the numerical results. The harmonic estimates are shown in the full abscissa ranges, as a reference, even in regions where the approximation is not expected to hold, because discrepancies give an idea of the departure from the harmonic response.

Figure 5c shows outcomes for a fixed $q<0$ ($q=-0.5$), corresponding to a vertical cut in the diagram of Fig. 5a. Sustained oscillations (i.e., $\bar{x} \rightarrow 0$) can emerge for $q<0$, when diffusion is weak, namely, for $D<D_c \simeq 0.0025$ (lilac colored region), where $D_c$ is obtained from $\lambda (k^\star )=0$. When *D* increases beyond this critical value, oscillations are damped with a finite characteristic length $\bar{x}$. For even larger values of *D*, oscillations completely disappear ($\bar{k}\rightarrow 0$). Note that the comparison between numerics and harmonic theory (symbols vs. dashed lines) is good close to the pattern transition point $D_c$, where the response peak is sharp (large *Q*). Despite the lack of agreement for larger *D*, the harmonic approximation qualitatively works with a shift of the transition from attenuated oscillations to exponential decay.

Figure 5d (which corresponds to vertical cut at $q=0.5$ in the diagram of Fig. 5a) shows the corresponding results for a fixed $q>0$ ($q=0.5$), which is characterized by the absence of sustained patterns.

Above $D\simeq 0.02$, the response $\tilde{R}(k)$ is unimodal, a bell-shaped curve that resembles the harmonic response, as in the case $D=0.2$ (black lines) in Fig. 6, producing a good agreement between harmonic and numerical results, despite being far from the large-*Q* limit. However, for smaller values of *D*, the profile is multi-peaked, and not even $k^\star$ predicts the observed mode, indicating that the harmonic approximation does not hold, as for $D=2\times 10^4$ (red lines) in Fig. 6. In this regime, it is crucial to analyze the response function in terms of complex poles in order to extract the dominant mode and its decay.

Figure 5b displays $\bar{k}$ and $\bar{x}$ as a function of *q*, for a fixed value of the diffusion coefficient ($D=10^{-3}$), corresponding to a horizontal cut in Fig. 5a. Recall that, the smaller the value of *q*, the more confined is the interaction (thus, the larger is $\bar{x}$). For $q < q_c \approx -0.093$ there are sustained oscillations ($\bar{x} \rightarrow \infty$). Above $q_c$, oscillations decay, which is indicated by the transition of $1/\bar{x}$ from null to finite values. Again, near this transition, the harmonic approximation works well, but, far from the critical point, it fails, as noticed above $q\simeq 0.2$, where there is a strong mismatch between the main mode given by the harmonic approximation and the numerical one. Also in this case, a small hump in the response function represents the dominant mode, as predicted by the analysis of the complex poles of $\tilde{R}$.

### Two-dimensional landscapes

In this section, we show results of simulations for relevant 2D scenarios, verifying that the picture of induced oscillations described up to now for 1D also holds in 2D.

**Figure 7:**
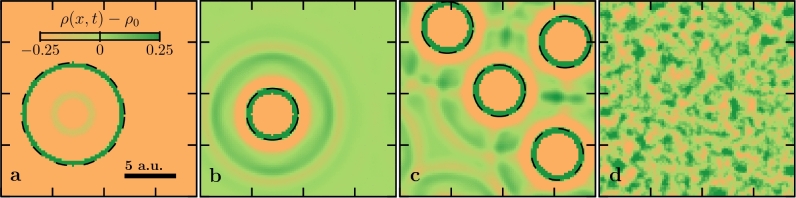
Long-time spatial distribution in 2D. Simulated scenarios: (**a**) a circular region (with radius 5 a.u., highlighted with a black dashed boundary) where the growth rate is positive, *a* (in a strong negative background $a-A$); (**b**) a circular region (with radius 2.5 a.u., highlighted with a black dashed boundary) where the growth is strongly negative $a-A$ (while outside, it is positive, *a*); (**c**) four regions with negative growth rates $a-A$ (in a positive background, *a*); (**d**) time-independent random landscape (where each spatial cell is assigned a growth rate uniformly distributed in [0.5*a*, 1.5*a*]). In all cases the interaction kernel is $\gamma _q$, with $\ell =2$ and $q=0.5$, $D=10^{-3}$, $a=b=1$ and $A=10$ . Colors show the deviation from the homogeneous state $\rho (x,t)-\rho _0$ (where $\rho _0 = 1$ for the chosen values of the parameters). For numerical integration, a pseudo-spectral method^45^ was used with $\Delta x = 0.2$ and $\Delta t = 10^{-3}$.

Snapshots of simulations for different 2D landscapes are presented in Fig. 7: a refuge (a), a defect (b), multiple defects (c) and spatial randomness (d) where many spatial scales are present. It is worth remarking that, in 2D, for the kernel $\gamma _q$, patterns only appear in homogeneous landscapes if $q<q_c \simeq 0.25$ (i.e., if $\lambda (k^\star )>0$). Thus, in all the cases of Fig. 7 (using $q=0.5$) we would not find oscillations if the landscape were homogeneous. In Fig. 7, we see that for 2D the same picture as in 1D is found: decaying oscillations appear near landscape disturbances with a clear wavenumber and decay length. The linear response approach presented in Semi-infinite habitat section can straightforwardly be extended to 2D. Figure 7a–c shows the case in which defects either increase or decrease the population growth rate. This can be promoted by ecosystem engineers such as termites^38^. Figure 7d shows a case where the landscape is random (in space, but time-independent). This situation, investigated in many previous studies^20,36^, produces a pattern that is noisy but has a dominant wavelength, which is related to $\ell$. Furthermore, although there is not a clear identification of decay length from pattern observation, the linear theory would allow one to estimate the characteristic spatial correlation length from the width of the Fourier spectrum.

## Inferring information about the interactions

**Figure 8:**
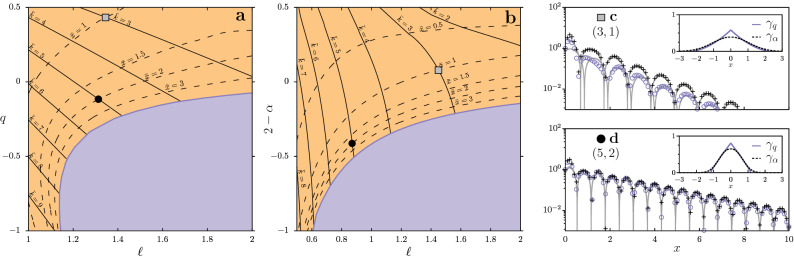
Determination of oscillation wavenumber, $\bar{k}$, and decay length, $\bar{x}$. Contour lines for fixed wavenumber (solid lines) and decay lengths (dashed lines). Colors for different oscillatory regimes are applied to the background as in previous figures. We considered the interaction kernel $\gamma _q$, given by Eq. (4), in (**a**) and $\gamma _\alpha \equiv N_\alpha ^{-1}e^{-|x/\ell |^\alpha }$ in (**b**). The remaining parameters are $D=10^{-3}$ and $a=b=1$. The highlighted points correspond to $(\bar{k},\bar{x})=(3,1)$ (gray square) and $(\bar{k},\bar{x})=(5,2)$ (black circle). (**c**), (**d**) Oscillations produced by the kernels shown in the respective insets (case $\gamma _q$ in purple circles and $\gamma _\alpha$ in black crosses). For both panels, the inset shows the kernels $\gamma _q$ (solid purple) and $\gamma _\alpha$ (dashed black) obtained from the oscillation’s parameters for the highlighted points: (**c**) $(\ell ,q) = (1.345,0.433)$, $(\ell ,2-\alpha ) = (1.450,0.079)$ (gray squares), and (d) $(\ell ,q) = (1.313, -0.116)$, $(\ell ,2-\alpha ) = (0.871,-0.413)$ (black circles). The gray lines show a sinusoidal fit for each case, namely $\rho _H(x) = 1 + Be^{-x/\bar{x}}\sin (\bar{k}x + \phi )$, with mode, $\bar{k}$, and decay, $\bar{x}$, as predicted by the mapping, while *B* and $\phi$ were adjusted.

In this section, we extend the discussion about the mapping between kernel and oscillation parameters, showing how information about the interactions can be extracted from landscape-induced oscillations. For that purpose, using the theoretical predictions given by Eq. (10), we obtained the contour lines for certain wavelengths $\bar{k}$ and decay lengths $\bar{x}$, in the space of the kernel parameters, as shown in the plane $(q,\ell )$ of Fig. 8a, for the kernel $\gamma _q$: $\bar{k}(\ell ,q) = \text {constant}$, and $\bar{x}(\ell ,q) = \text {constant}$.

These contour lines depend both on $\ell$ and *q*. However, while $\bar{k}$ is strongly controlled by the interaction scale, $\ell$, $\bar{x}$ is more closely related to the shape parameter *q*. As a consequence, there is a crossing of the lines that uniquely identifies the kernel properties. Of course, this is possible for the decaying-oscillation phase (orange region), in which oscillations have a well-defined $\bar{k}$ and $\bar{x}$. For the sustained-oscillation ($\bar{x}\rightarrow \infty$) and the exponential relaxation ($\bar{k}=0$) phases, the stationary distribution does not carry sufficient information to infer the specific values of *q* and $\ell$ (in the perspective of the linear theory).

For comparison, in Fig. 8b, we perform the same analysis considering the stretched-exponential kernel, $\gamma _\alpha (x) \equiv N_\alpha ^{-1} \exp (-|x/\ell |^\alpha )$, where $N_\alpha$ is the normalization factor. Likewise $\gamma _q$, this kernel also allows us to contemplate changes in the range and shape of the competitive interactions. Since, for $\gamma _\alpha$, compactness increases with $\alpha$, and sustained spatial oscillations require $2-\alpha <0$^24^, in Fig. 8b, we show contour lines for the same values used in Fig. 8a, in the plane defined  by parameters $2-\alpha$ and $\ell$. Also, in this case, the results show that the identification of the kernel features is possible. Additional results using this kernel can be found in the Supplementary Information.

To test the inference procedure, we imagine the scenario in which spatial oscillations with certain values of $\bar{k}$ and $\bar{x}$ are observed. Then, assuming that the population distribution evolves according to Eq. (3), one proposes a generalized form for $\gamma$ (as one of the two discussed above) and extracts the kernel parameters from the $(\ell ,\beta ) \leftrightarrow (\bar{k},\bar{x})$ mapping (Figs. 8a,b), where $\beta$ represents either *q* or $2-\alpha$ (for $\gamma _q$ and $\gamma _\alpha$, respectively).

In Fig. 8c,d, we verify that, in fact, using the extracted kernel parameters, $(\ell ,\beta )$, numerical simulations produce spatial oscillation with the correspondent $(\bar{k},\bar{x})$ (black circles and gray squares). Moreover, in the insets of Fig. 8c,d, we compare the inferred kernel under the $\gamma _q$ and $\gamma _\alpha$ representations. Note that, regardless of the particular choice made for $\gamma$, both profiles have the same coarse-grained appearance, allowing to qualitatively access the characteristic length and compactness of the influence function. However, we stress that since the information provided by the theory is limited, it is not possible to infer exactly the form of $\gamma$ just by measuring $(\bar{k}, \bar{x})$ of the oscillations. To access the fine details about the kernel, improvements of this methodology could look for information encoded in the spatial transient and nonlinear effects that occur close to the interface.

## Final remarks

Heterogeneities can modify system stability conditions^31,35,36^, inducing the emergence of states that would not be present under homogeneous conditions. In the context of biological populations with the potential to develop spatial patterns, it would be interesting to establish if the conditions for the occurrence of pattern formation can be modified by the presence of environmental inhomogeneities. Also, it is natural to ask under which conditions or how heterogeneities can be used to help in the task of identifying details about microscopic interaction from the observation of the macroscopic patterns^39^.

The first question was considered by Page et al.^34^ in the context of two-species reaction–diffusion models undergoing a pattern formation instability of the Turing type. It was found that the range of parameters for which periodic solutions were possible was extended by the presence of a discontinuity in some system parameter. Here, we have addressed this issue in a model of a population of competing organisms. In particular, we have considered a nonlocal FKPP equation which includes reproduction, diffusion, and competition between individuals at a distance. Non-local competitive interactions can arise due to different mechanisms, as in root-mediated competition for water in vegetation^10,11,38^, and release or consumption of intermediate substances by the organisms^10,14,15,19^. In all cases, the fact that competitive interactions are nonlocal plays a major role in the spatial organization of the population. In particular, pattern formation can occur in a manner related to the Turing case, although in the FKPP approach a single species is explicitly modeled, with competitive interactions effectively captured by the influence function, $\gamma$. The kernel family $\gamma _q$ was chosen in the examples because it allows shapes of different compactness. But results for another important class, the stretched exponential family, were presented in Supplementary Information. A necessary condition for the development of stable spatially periodic patterns starting from a uniform solution is that the interaction profile is sufficiently compact, meaning sub-triangular ($q < 0$), for the *q*-exponential (see Fig. 2a), or platykurtic ($\alpha > 2$), for the stretched exponential family (see Fig. S1).

Previous work already showed that these conditions become less strict when the initial condition contains sharp changes: propagating-front solutions of the nonlocal FKPP equation develop oscillatory patterns in cases when the influence function does not satisfy the above compactness condition^20,46^. Here, we have investigated how the above scenarios are modified by an abrupt change in the ecological landscape. We have seen that modes which are suppressed in the homogeneous-landscape case are activated by the interface, producing decaying oscillations. Activation of modes have been observed in particular realizations of the nonlocal FKPP triggered by random^20,33^ heterogeneities. In these cases, the variation of the equation parameters extends to all the space, generating a noisy pattern that has a clear dominant wavelength (see Fig. 7d). A localized perturbation (like the interface we consider) is more useful because it puts into evidence, besides the dominant wavenumber, also the decay length (see Fig. 7b), which reflects how a perturbation spreads in the system. When the landscape variation occurs in all points of space, the decay length is blurred. The implication of these results (alike in a multispecies reaction–diffusion situation^34^) is that the range of parameters for which spatial structures can occur in biological populations can be much larger than superficially expected.

Deepening further into the interplay between nonlocality and environmental heterogeneity, we have shown here that the presence of heterogeneities can reveal information about interaction scales that would otherwise be hidden. This is possible due to the existence, once a functional form for the influence function is fixed, of a one-to-one correspondence between the parameters of the competitive interactions (shape and range) and the landscape-induced oscillation features (wavenumber and decay length). So, the natural or artificial interposition of an interface can act as a lens that allows us to see what is veiled in a homogeneous landscape. This might be particularly useful in situations where the details of the long-range influence are not perfectly clear, depending on a complex combination of ecological factors. Although the approach provides the coarse-grained profile of the influence function, without distinguishing other fine details, our results allow to take a step forward in the direction of understanding the connection between interactions at the individual level and the emergent macroscopic patterns^39^.

The numerical results were perfectly predicted by the analysis of the poles of the system response function. Additionally, we have presented a harmonic approximation that has limitations but provides a more direct insight. The analytical results were obtained for general forms of the landscape $\Psi (x)$ and can be used to understand the effects of arbitrary heterogeneous landscapes, like the multiple and random cases shown in Fig. 7. But we have focused on the case of a single interface because of its above-discussed features.

It is worth remarking that, due to computational cost, we compared theoretical predictions with numerical simulations mostly for 1D, but we showed also similar outcomes in some 2D environments. Lastly, it is also interesting to remark that the reported results can reach contexts beyond population dynamics, since the interplay between nonlocality and heterogeneity is found in diverse systems.

## Supplementary Information

Supplementary Information.
