# Supplementary material for: Landscape-induced spatial oscillations in population dynamics
Source: Sci Rep. 2021 Feb 10;11:3470. doi: 10.1038/s41598-021-82344-8 (PMC7876042; doi:10.1038/s41598-021-82344-8)
Supplement: Supplementary file 1 — Supplementary Information. [file 41598_2021_82344_MOESM1_ESM.pdf]

Supplementary information: Landscape-induced spatial oscillations in population dynamics

Vivian Dornelas, Eduardo H. Colombo, Cristóbal López, Emilio Hernández-García, Celia Anteneodo

Numerical method

The numerical solution of the one-dimensional generalized FKPP equation presented in Eq. (3) of the main text was obtained by means of a finite-difference forward-time-centered-space (FTCS) scheme, implemented in C language.

For the spatial grid, we consider equally spaced mesh points $x_i = i\Delta x$, with integer i and grid space Δx. At each mesh point x_i, the density at time t_j, $\rho_j^i = \rho(x_i, t_j)$, evolves according to an explicit scheme with fixed time step Δt, such that $t_j = j\Delta t$ with integer j. For the spatial discretization of the second derivative, we used the centered form $\partial_{xx}\rho_j^i = (-\rho_j^{i+2} + 16\rho_j^{i+1} - 30\rho_j^i + 16\rho_j^{i-1} - \rho_j^{i-2})/(12\Delta x^2)$, and for the integration of the nonlocal competition term we used the trapezoidal rule. Lastly, a fourth-order Runge-Kutta approximation for the new values ρ_i^{j+1} was used[1], which improves the stability domain in comparison with the Euler method. Typical values used for spatial and temporal discretization, respectively, are $\Delta x = 0.05$ and $\Delta t \in [10^{-2}, 10^{-4}]$ (depending on D), leading to an estimated relative error smaller than 10^{-4}.

For the initial preparation of the system, we applied a small random perturbation around the homogeneous solution $\rho_0 = a/b$, drawing a random number ξ^i uniform in $(-\varepsilon, \varepsilon)$, with $\varepsilon \ll \rho_0$, for each mesh point x_i, such that, $\rho_0^i = \rho_0 + \xi^i$.

The boundary conditions for each configuration are described below (in Section *Boundary conditions*) and in Section *Stationarity condition*, we show the stop criterion used to determine stationarity.

The two-dimensional simulations in Fig. 7 of the main text were performed using the pseudospectral algorithm of Ref. [46], with $\Delta t = 10^{-3}$ and $\Delta x = 0.2$.

Boundary conditions

Different boundary conditions were adopted along the main text:

(i) For the homogeneous landscape (Fig. 1a) and for (finite) refuge case (Fig. 3), we used periodic boundary conditions.

(ii) For the semi-infinite habitat (e.g., in Fig. S1, with growth rate a in the region $x \geq 0$, and with growth rate $a - A$, otherwise), the integration was performed in a grid with $-L \leq x \leq L$, using $L = 100$, much larger than oscillation length-scales, under the constraints $\rho(x \leq -L) = (a - A)/b$ and $\rho(x \geq L) = \rho_0 = a/b$.

(iii) For the semi-infinite habitat with strong harmful conditions, $A \to \infty$ (see profiles in Figs. 1b, 4, 8c, 8d), the integration

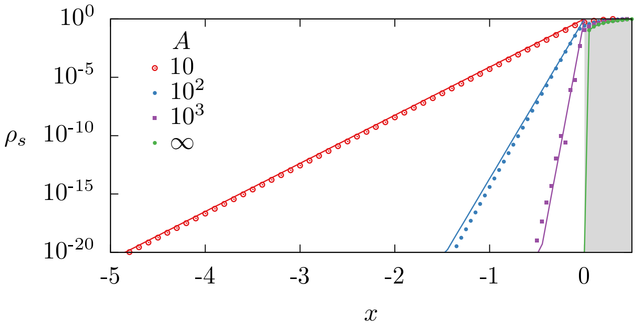

Figure S1. Exponential decay from the interface (for $A >> a$). Numerical integration for the semi-infinite habitat with very strong harmful conditions outside the habitat (symbols), for different values of A indicated in the legend. The grey region (at $x > 0$), represents the refuge near the interface. The solid lines represent the exponential decay predicted by $\rho(x \leq 0) \sim e^{\sqrt{(A-a)/D}x}$. The influence kernel is γ_q with $q = 0.1$ and $\ell = 2$, $D = 0.1$ and $a = b = 1$.

was performed in a grid with $0 \leq x \leq L$, using $L = 100$, under the conditions $\rho(x < 0) = 0$ and $\rho(x \geq L) = \rho_0 = a/b$. This choice is justified by the fact that in the limit $A \to \infty$, the density outside the refuge vanishes, as shown in Fig. S1.

Stationarity criterion

Figure S2a shows the population density $\rho(x,t)$ vs. x at different instants of time t, obtained from the integration of Eq. (3) for a semi-infinite refuge with strong harmful conditions outside ($A \to \infty$). As time passes, the profile progressively attains a stationary form, $\rho(x,\infty)$. This limiting value can be estimated by noting that the relative difference (discrepancy) between the profiles at different instants decays exponentially with time. In Fig. S2b, the discrepancy $|1-\rho(x_i,t)/\rho(x_i,\infty)|$, for selected mesh points x_i is displayed, showing exponential convergence. The final simulation time was chosen such that the discrepancy is smaller than 10^{-4} for all the mesh points in the interval of interest.

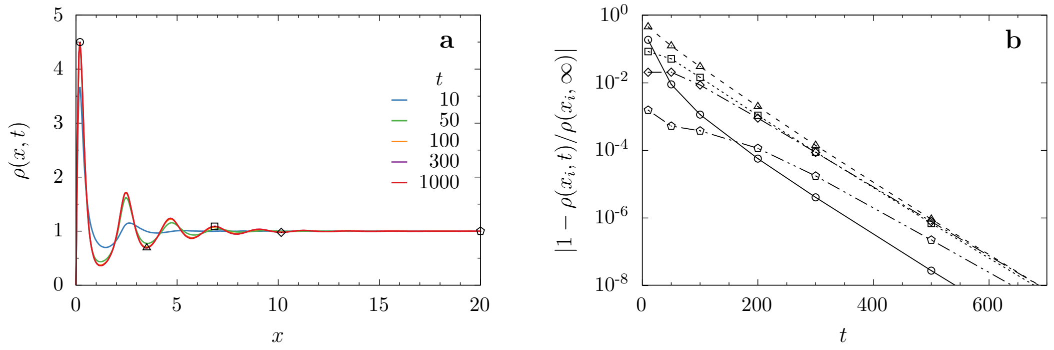

Figure S2. Approaching the stationary profile. (a) Decaying oscillations in population density in a semi-infinite refuge with strong harmful conditions outside ($A \to \infty$), at different times indicated in the figure. Eq. (3) of the main text, using γ_q with $q = 0.1$ and $\ell = 2$, and $D = 10^{-3}$, was numerically integrated using $\Delta x = 0.05$ and $\Delta t = 0.01$. (b) Relative difference $|1-\rho(x_i,t)/\rho(x_i,\infty)|$ is plotted vs. time, for the values of x_i selected in panel (a), identified by the same symbols, showing that stationary values are exponentially approached.

Results for the stretched exponential kernel

We consider, as a second class of kernels, the stretched exponential family,

$$\gamma_\alpha(x) = \frac{e^{-(|x|/\ell)^\alpha}}{2\ell\Gamma(1+1/\alpha)}\,, \tag{1}$$

with $\alpha > 0$ (to guarantee normalization). When $\alpha = 1$, it produces the double exponential kernel, whose Fourier transform is $\tilde{\gamma}_1(k) = \frac{1}{1+k^2\ell^2}$. It includes the Gaussian ($\alpha = 2$), whose Fourier transform is $\tilde{\gamma}_2(k) = e^{-k^2\ell^2/4}$. And it also reproduces the top-hat kernel in the limit $\alpha \to \infty$, that has $\tilde{\gamma}_\infty(k) = \sin(k\ell)/(k\ell)$.

In Fig. S3, we show the mode growth rate, $\lambda(k)$, for three values of α. And in Fig. S4a, we show the phase diagram. In Fig. S4b, we characterize the profiles as a function of parameter $2-\alpha$, for $D = 10^{-3}$. All these results qualitatively resemble those produced in the main text for kernel γ_q (Fig. 2 and 5 of the main text).

References

1. Press, William H and Teukolsky, Saul A and Vetterling, William T and Flannery, Brian P, *Numerical Recipes in C* (Cambridge university press, 2007).

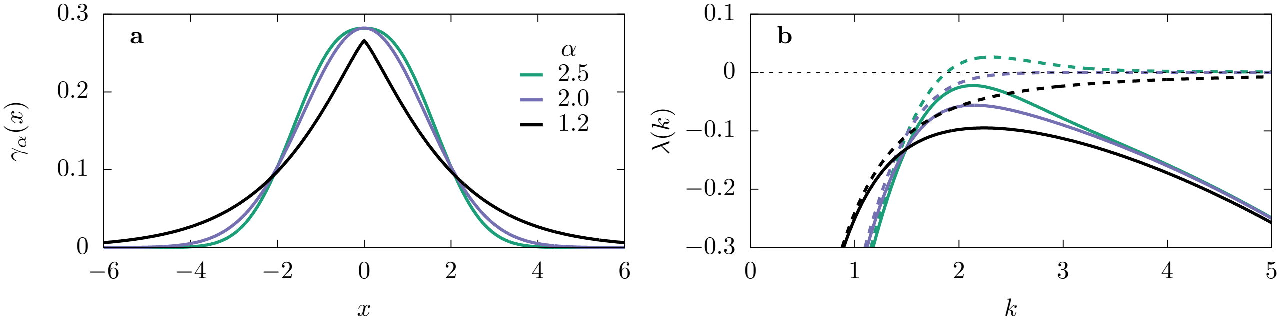

Figure S3. Interaction kernel and mode stability in a homogeneous medium. (a) $\gamma_\alpha(x)$, defined in Eq. (1), for the values of α indicated on the figure, and $\ell = 2$. (b) Mode growth rate $\lambda(k)$, for $a = b = 1$, with $D = 0$ (dashed lines) and $D = 10^{-2}$ (solid lines), corresponding to the values of α plotted in (a). The case $\alpha = 2$ is the critical one, for which the maximal value of $\lambda(k)$ at finite k is zero when $D = 0$.

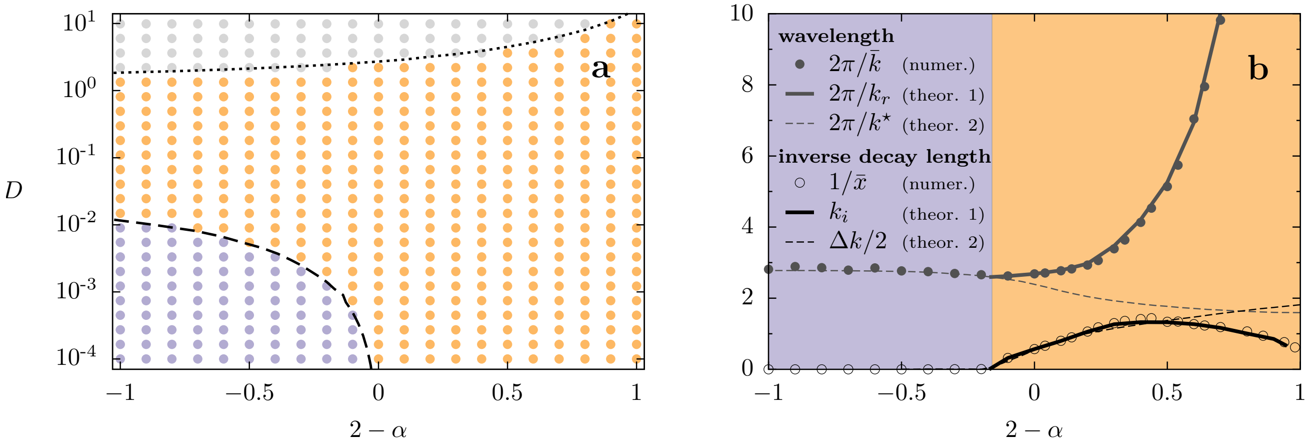

Figure S4. Phase diagram and characteristics of the stationary profiles as a function of shape parameter $2-\alpha$, for kernel $\gamma_\alpha(x)$ with $\ell = 2$. The remaining conditions and conventions are as in Fig. 5 of the main text. In panel b, $D = 10^{-3}$.
